# Prolactin and vasoinhibin are endogenous players in diabetic retinopathy revisited

**DOI:** 10.3389/fendo.2022.994898

**Published:** 2022-09-09

**Authors:** Jakob Triebel, Thomas Bertsch, Carmen Clapp

**Affiliations:** ^1^ Institute for Clinical Chemistry, Laboratory Medicine and Transfusion Medicine, General Hospital Nuremberg and Paracelsus Medical University, Nuremberg, Germany; ^2^ Instituto de Neurobiología, Universidad Nacional Autónoma de México (UNAM), Campus UNAM-Juriquilla, Querétaro, Mexico

**Keywords:** vasoinhibin, PRL, diabetic retinopathy, diabetic macular edema, diabetes, levosulpiride

## Abstract

Diabetic retinopathy (DR) and diabetic macular edema (DME) are major causes for visual loss in adults. Nearly half of the world’s population with diabetes has some degree of DR, and DME is a major cause of visual impairment in these patients. Severe vision loss occurs because of tractional retinal detachment due to retinal neovascularization, but the most common cause of moderate vision loss occurs in DME where excessive vascular permeability leads to the exudation and accumulation of extracellular fluid and proteins in the macula. Metabolic control stands as an effective mean for controlling retinal vascular alterations in some but not all patients with diabetes, and the search of other modifiable factors affecting the risk for diabetic microvascular complications is warranted. Prolactin (PRL) and its proteolytic fragment, vasoinhibin, have emerged as endogenous regulators of retinal blood vessels. PRL acquires antiangiogenic and anti-vasopermeability properties after undergoing proteolytic cleavage to vasoinhibin, which helps restrict the vascularization of ocular organs and, upon disruption, promotes retinal vascular alterations characteristic of DR and DME. Evidence is linking PRL (and other pituitary hormones) and vasoinhibin to DR and recent preclinical and clinical evidence supports their translation into novel therapeutic approaches.

## Diabetic Retinopathy is a common cause of vision loss and blindness

Most patients with longstanding diabetes mellitus develop microvascular complications of diabetes, namely nephropathy, neuropathy, and retinopathy. DR is a highly specific neurovascular complication of diabetes and is the most frequent cause of new blindness among adults aged 20-74 years in developed countries (1, 2). DR advances from mild nonproliferative abnormalities with increased vasopermeability and microaneurysms to moderate and severe stages characterized by the growth of new blood vessels in the retina and the posterior surface of the vitreous. Fibrous tissue may exert tension on the retina and cause retinal detachment. The new blood vessels may bleed and cause preretinal and vitreous hemorrhage. A macular edema causing central vision impairment may occur because of increased vasopermeability and capillary nonperfusion (3). Major risk factors include the duration of diabetes, HbA1c levels, and blood pressure (3, 4). The onset of puberty and pregnancy increase the risk of progression of DR. Tertiary prevention of DR includes laser photocoagulation for proliferative diabetic retinopathy (PDR), anti-VEGF therapy for DME and PDR, and vitrectomy in advanced DR (5). Various pathophysiological and pathobiochemical pathways directly linked to chronic hyperglycemia which lead to a disorganization and breakdown of the blood-retinal-barrier are involved in the manifestation of DR and DME, including an activation of protein kinase C (6) and the accumulation of advanced glycation end products (7). However, there are patient populations with type 1 diabetes of extreme duration who do not develop diabetic complications and appear to be protected by unknown factors (8, 9). This contrasts with other studies, which usually report that >90% of patients with type 1 diabetes will eventually develop retinopathy (10). Also, there was a lack of association between glycemic control and prevalence of reported microvascular complications (11). Consistently, the total glycemic exposure (A1C and duration of diabetes) explained only 11% of the variation in risk in the Diabetes Control and Complications Trial (DCCT) cohort, where retinopathy progression was studied in conventional and intensive treatment groups (12). It is thus acknowledged that significant numbers of patients with diabetes can live without severe complications, likely due to factors that can neutralize the adverse effects of hyperglycemia or other unknown protective factors which prevent the development of diabetic complications (11). Hormonal factors are predisposed to confer protective effects against microvascular complications through their effects on organ function, repair and maintenance of homeostasis, the control of growth, and their capacity to adapt their levels and action in response to demand or to pathologic stimuli. The investigation of pituitary hormones is therefore warranted.

## Pituitary infarction revealed an involvement of pituitary hormones in diabetic retinopathy

A role of pituitary hormones in the etiopathology of DR emerged soon after the observation that infarction or insufficiency of the anterior lobe of the pituitary, can result in hypoglycemia and high sensitivity to administered insulin, known as the Houssay-Biasotti phenomenon. In fact, infarction, or insufficiency of the pituitary gland, also known as Simmond’s disease, can lead to terminal hypoglycemia, as reported in a series of early case studies (13, 14). Pituitary infarction can also occur after severe peri- or postpartum hemorrhage, as described by Sheehan (Sheehan’s syndrome). In all instances, examples of cessation or regression of diabetic retinopathy was observed. Soon thereafter, pituitary ablations, stalk sections, and destruction by irradiation were introduced for treating diabetic retinopathy but became obsolete in the face of the harmful effects that were associated with these procedures and the following anterior pituitary insufficiency. The beneficial effects of pituitary insufficiency were attributed to the cessation of growth hormone secretion and consecutively lower insulin-like growth factor I (IGF-I) levels, however, the overall resumé of repeated cross-sectional, longitudinal, and prospective studies on the relationship between circulating IGF-I levels and DR did not establish a clear role for the GH/IGF-I axis (15). Patients with acromegaly and diabetes mellitus do not have a higher prevalence of DR (16) and patients with diabetes and congenital IGF-I deficiency (Laron syndrome) or GH gene deletion can develop DR (17, 18). Disparate data are available on circulating IGF-I levels and DR progression during pregnancy, with studies finding or not finding an association of IGF-I levels with DR during pregnancy (19, 20). On the other hand, it is known that an acute reduction of chronic hyperglycemia can accelerate DR, and that this deterioration is preceded by an upregulation of serum IGF-I (21). Both, GH, and IGF-I are present in the vitreous and the levels of IGF-I are higher in the vitreous of patients with retinal neovascularization (22, 23). Mechanistically, IGF-I has mitogenic and differentiating effects on cultured retinal endothelial cells (24) and on retinal capillaries (25), and can induce neovascularization in the avascular rabbit cornea (26). IGF-I and its receptor, as well as IGF binding proteins are distributed throughout the retina, and IGF-I mRNA has been detected in the ganglion cell layer, the inner nuclear layer and in the outer limiting membrane (27, 28). The total IGF-I distribution in ocular tissues is therefore a combination of local expression and systemic uptake. Altogether, the contribution of local and circulating IGF-I in diabetic retinopathy remains to be understood, can be interpreted as rather “permissive” than causal (17) and therapeutic interventions into the GH/IGF-I axis did not yield sufficient evidence in clinical studies to be considered in the current treatment recommendations for DR (5). Attesting to the heterogeneity and variation in pathomechanisms of proliferative retinopathies across the lifespan, ample evidence demonstrates the key role of IGF-I in retinopathy of prematurity (29–32).

## Circulating PRL levels change in diabetes

Another pituitary hormone which attracted attention in respect to its involvement in DR is PRL. Not long after the radioimmunoassay for PRL became available, which allowed the measurement of circulating PRL concentrations (33, 34), PRL was evaluated in patients without DR and DR at various stages. Early reports found higher PRL levels in patients with diabetes but without severe DR and hypothesized about the potential function of PRL as a protective factor in DR, and about some potential treatment based on the stimulation of PRL secretion (35, 36). Indeed, pituitary stalk section results in minimized GH secretion with subsequent decline of IGF-I levels but result in higher PRL-secretion due to a disinhibition of lactotroph PRL secretion by the disruption of dopamine transport through the pituitary stalk (37). The beneficial effects of pituitary stalk sections could therefore have been not only due to the reduction of IGF-I levels, but also due to an increase in circulating PRL. Comparable with IGF-I levels, various results were reported in which the association of PRL levels with DR presence and severity was not confirmed (38–41). A mechanism of action for protective effects of PRL levels was also missing. PRL exerts a diverse array of biological functions beyond its essential role in lactation (42–44), a fact which has received little attention in clinical medicine in the past, where the relevance of PRL is acknowledged in prolactinoma and secondary amenorrhea. Regarding diabetes and its complications, there is a new trend towards the recognition of PRL as an important metabolic hormone, directly involved in beta-cell function and survival, and the regulation of insulin sensitivity and resistance, respectively (45). Higher PRL levels are associated with higher insulin sensitivity and a lower incidence of type 2 diabetes mellitus, which led to a re-evaluation of current thresholds for normal PRL levels and hyperprolactinemia (45). It was proposed to re-define the interpretation of PRL levels beyond the upper threshold of 25 ng/ml where a homeostatic functionally increased transient hyperprolactinemia (homeoFIT) can be assumed, the suggested term for an elevation of PRL levels which may constitute a physiological response to increased metabolic demand (reviewed in ref. 45).

## The PRL/vasoinhibin axis controls ocular angiogenesis and vascular function

A new perspective on the role of PRL in DR began to evolve when the antiangiogenic effects of an enzymatically cleaved 16 kDa N-terminal fragment of human PRL were discovered (46), and a direct pathophysiological implication towards the regulation of blood vessel growth emerged. It became evident that the 16 kDa N-terminal fragment is not the only fragment with antiangiogenic effects, and that multiple isoforms with a large variation in molecular mass exist, their size being determined by the PRL-cleaving enzyme and its cleavage site location within the PRL molecule. The isoforms were collectively called vasoinhibin (47–49), including similar proteins generated by the proteolytic cleavage of GH and placental lactogen (PL) (50, 51). A strong role of vasoinhibin as a regulator of ocular angiogenesis and vascular function evolved, and with reference to existing reviews (52–55), and 11 years after PRL and vasoinhibin were first portrayed as endogenous players in DR (56), the following discussion will focus on key principles and significant developments in the recent years (**Table 1**). The new understanding of circulating PRL levels in terms of homeoFIT-levels is relevant when considering the role of PRL and vasoinhibin in DR, as in partial disagreement to the early studies between 1970 and 1985, there appeared to be an association between circulating PRL levels and DR, reported by Arnold et al. in 2010 (62). The PRL levels were higher in patients with diabetes and no retinopathy (compared to healthy controls) and higher in patients with diabetes and non-proliferative DR than in patients with PDR (62). The PRL levels in the patients with diabetes were above the conventional threshold of 25 ng/ml, and therefore in the homeoFIT-range. In addition to answering to increased metabolic demand, PRL levels in the homeoFIT-range may also, through their proteolytic conversion to vasoinhibin, contribute to control the function and growth of ocular blood vessels. Interestingly, uncleaved PRL is protective in the retina and required for maintaining retinal functionality in mice during aging and has potential therapeutic value against age-related retinal disorders (68, 69). Short PRL isoforms are expressed in the canine retina undergoing retinal degeneration (70). A clinical study in patients with a prolactinoma using optical coherence tomography revealed a reduced thickness of the chorioretinal layers in patients with prolactinoma compared to controls (71). Patients with DR have a higher renal elimination of PRL (72) and the circulating levels of vasoinhibin are reduced in patients with DR (63).

**Table 1:** Landmark original research articles and reviews highlighting the involvement of the prolactin/vasoinhibin axis in diabetic retinopathy.

Brief description	Year	Ref.
ORIGINAL RESEARCH ARTICLES	
Sulpiride-induced hyperprolactinaemia inhibits the diabetes- and VEGF-mediated increase in retinal vasopermeability by promoting the intraocular conversion of endogenous PRL to vasoinhibin	2022	(57)
Levosulpiride increases the levels of PRL in the vitreous of PDR patients and promotes its MMP-mediated conversion to vasoinhibin, which can inhibit angiogenesis in DR	2020	(58)
Study protocol of a prospective, randomized, double-blind, placebo-controlled trial enrolling male and female patients with type 2 diabetes having DME, randomized to receive placebo or levosulpiride	2018	(59)
AAV2 vasoinhibin vector decreases retinal microvascular abnormalities in rats	2016	(60)
AAV2-vasoinhibin vector in rats prevents pathologic retinal vasopermeability and suggest it could have therapeutic value in patients with DR	2011	(61)
Circulating PRL influences the progression of DR after its intraocular conversion to vasoinhibin. Inducing hyperprolactinemia may represent a novel therapy against DR	2010	(62)
Patients with diabetes mellitus and DR have lower circulating levels of vasoinhibin, compared to healthy patients	2009	(63)
Vasoinhibin blocks retinal vasopermeability in diabetic rats and in response to intravitreous injection of VEGF or of vitreous from patients with DR	2008	(64)
Vasoinhibin is a natural inhibitor of angiogenesis in the retina	2005	(65)
Vasoinhibin is a natural inhibitor of corneal vascularization	1999	(66)
Speculations whether stimulating PRL-release in patients with DR might be benefitial	1976	(36)
**REVIEW ARTICLES**		
Pharmacological interventions into the prolactin/vasoinhibin axis for the treatment of diabetic retinopathy	2017	(52)
Introduction of the prolactin/vasoinhibin axis and its pathophysiological significance including DR	2015	(67)
Review of the regulation of blood vessel growth and function by vasoinhibin	2015	(53)
Portray and review of PRL and vasoinhibin as endogenous players in DR	2011	(56)
Introduction of vasoinhibin as a novel inhibitor of ocular angiogenesis	2008	(55)

The principle underlying vasoinhibin accumulation in the retina – or in other tissues – is that of an endocrine axis in which the levels of vasoinhibin are controlled by regulatory mechanisms at the hypothalamo-, the pituitary-, and the local level. The vasoinhibin levels depend on the availability and amount of secreted and circulating PRL (hypothalamo-pituitary level), and on the hypothalamo, pituitary, and peripheral tissue distribution and activities of PRL-cleaving proteases (local level). This hormonal axis was described as the PRL/vasoinhibin axis of which the vasculature is a major target tissue (53, 67). The cleavage sites in PRL through which vasoinhibin is generated are conserved in vertebrates (47, 67, 73) and high affinity cleavages sites evolved, most likely as a gain of function under positive selection, as a unique feature of higher primates (74). The cleavage of PRL to generate vasoinhibin occurs in the wider context of a hormone-metabolism junction, through which specifically cleaved hormones regulate essential functions to maintain homeostasis at the organismal, tissue, or organ levels (75, 76). The PRL/vasoinhibin axis contributes to maintaining corneal avascularity (66), restricts retinal vasculature (65), and is disrupted in retinopathy of prematurity (77, 78). In rodents, hyperprolactinemia leads to vasoinhibin accumulation in the retina and reduces both VEGF-induced and diabetes-induced retinal vasopermeability (57, 62, 64); an effect also demonstrated by vasoinhibin gene transfer which not only prevented (61) but also reversed (60) excessive retinal vasopermeability and oxygen-induced retinal angiogenesis (79).

The bioactive site in vasoinhibin, through which the antiangiogenic and antivasopermeability effects of the molecule are mediated, is a short, conserved three-residue motif consisting of residues His46-Gly47-Arg48 which becomes active after the proteolytic cleavage of PRL to vasoinhibin (80). Molecular dynamics simulations predicted the three-dimensional structure of vasoinhibin comprising a three-helix bundle with a tendency to form dimers or multimers, which also complicated the experimental resolution of the vasoinhibin three-dimensional structure (73, 81, 82). Vasoinhibin signals through various binding partners such as a specific high affinity binding site on endothelial cells (83), integrin alpha5 beta1 (84), or plasminogen activator inhibitor 1, urokinase, and urokinase receptor multicomponent complex (85) to trigger intracellular signaling pathways that result in its effects on endothelial cells but a classical hormone receptor has not been identified. The circulating levels of vasoinhibin are unknown due to the absence of a quantitative vasoinhibin assay for human serum, which is why immunoprecipitation followed by SDS-PAGE and Western blotting is still the only more frequently used method for the evaluation of vasoinhibin in clinical samples (77). Alternative methods using a lab-on-a chip technology or mass spectrometry were reported (63, 86, 87) but did not establish themselves thereafter. The lack of monoclonal anti-vasoinhibin antibodies able to discriminate between PRL and vasoinhibin prevented attempts to develop a sandwich enzyme-linked immunosorbent assay (ELISA). Fortunately, monoclonal antibodies were recently developed, and their evaluation for an ELISA by which the levels of vasoinhibin could be quantified is underway (88). However, Western blot evaluation of vasoinhibin in clinical samples is supported by the measurement of its antiangiogenic properties in the presence or absence of anti-PRL antibodies that neutralize vasoinhibin action (58, 89).

## A clinical trial investigates the elevation of PRL-levels in patients with diabetic retinopathy

Increased, hypoxia-driven expression of VEGF, produced by the retinal pigment epithelium, by endothelial cells, pericytes and other retinal cells, with consecutive enrichment in the retina and vitreous is a major driver of DME and PDR as it contributes to rupturing the blood-retinal barrier and induces angiogenesis which results in pathological neovascularization. The healthy vitreous is one of the few naturally avascular structures but is invaded by blood vessels in PDR. Not only the elevation of growth factors facilitates its invasion by neovessels, the impaired production or insufficient upregulation of natural blood vessel inhibitors responsible for maintaining the avascular state of the vitreous are relevant as well (90). The healthy vitreous humor as such is antiangiogenic and inhibits tumor neovascularization (91), and angiogenesis in various other models, for example the retinal-extract induced angiogenesis in the chick chorioallantoic membrane (CAM) assay (92).

**Figure 1:**
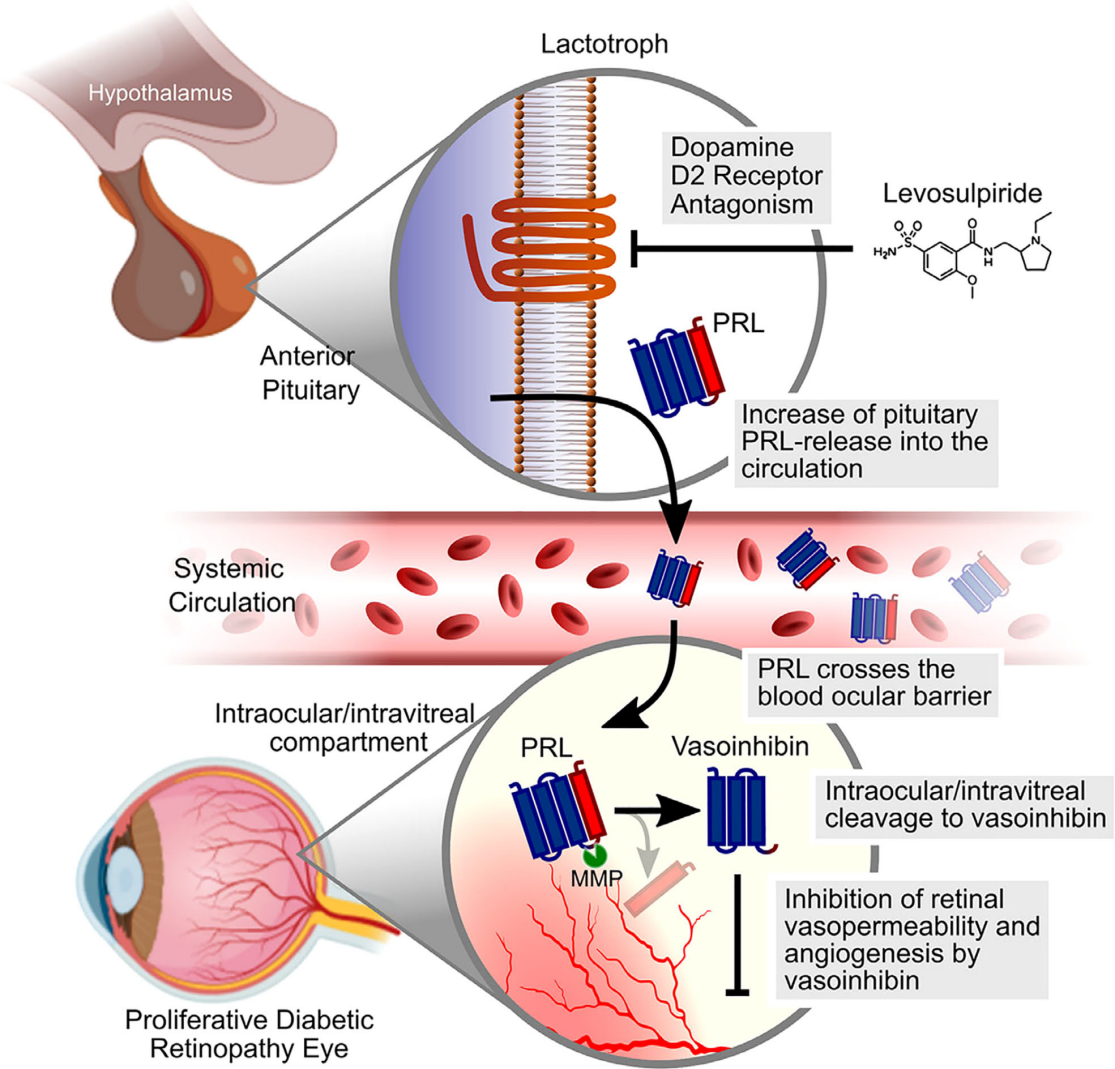
Schematic representation of the mechanism by which levosulpiride therapy could limit the progression of DME and DR. Levosulpiride, a dopamine D2 receptor antagonist, blocks dopamine D2 receptors located in the membrane of anterior pituitary cells that produce PRL (lactotrophs). Given that hypothalamic dopamine inhibits the release of PRL, levosulpiride leads to high levels of PRL in the circulation (hyperprolactinemia) which, in turn, favor PRL penetration across the blood–ocular barrier. MMPs in the intraocular/vitreous compartment cleave PRL to vasoinhibin, which can reduce retinal vasopermeability and angiogenesis in DME and DR. Scheme was partly created with Biorender.com. The original figure was published by Nunez-Amaro et al. (58) under the Creative Commons Attribution-Non-Commercial-NoDerivatives 4.0 International License (https://creativecommons.org/licenses/by-nc-nd/4.0/). The figure was not modified.

As mentioned, hyperprolactinemia leads to vasoinhibin accumulation in the retina of rats and prevents and reverses diabetes-induced blood retinal barrier breakdown and ischemia-induced angiogenesis by inhibiting vasopermeability and by targeting the retinal pigment epithelial cells in the outer blood retinal barrier (62, 93). These insights triggered the development of a randomized clinical trial, in which levosulpiride is evaluated as a medical treatment in patients with PDR and DME (59) (**Figure 1**). Levosulpiride is a dopamine D2 receptor blocker which is used as a prokinetic drug in patients with diabetic gastroparesis, where enteric inhibitory dopaminergic D2 receptor antagonism can have prokinetic effects. At the pituitary level D2 receptor antagonism with levosulpiride evokes hyperprolactinemia (94). One arm of the clinical study includes patients with PDR undergoing vitrectomy, with and without prior treatment with levosulpiride and subsequent laboratory evaluation of the vitreous fluid. Levosulpiride treatment increased PRL and vasoinhibin in the vitreous, and the vitreous from levosulpiride-treated patients with PDR, but not from placebo-treated patients with PDR, inhibited the basic fibroblast growth factor (bFGF) and VEGF-induced proliferation of endothelial cells in culture (58). The conversion of PRL to vasoinhibin was mediated by matrix metalloprotease (MMP) present in the vitreous fluid and was higher in patients without diabetes than in patients with PDR (58). This result is the first partial outcome of the clinical study which provided a proof-of-concept that treatment with levosulpiride is appropriate to elevate intraocular PRL and vasoinhibin levels. Further proof-of-concept was shown by an *in vivo* study in rats with streptozotocin-induced diabetes, in which racemic sulpiride increased ocular vasoinhibin levels and inhibited retinal hypervasopermeability (57). The other arms of the trial that also comprise patients with DME are awaiting completion and the publication of the results are expected soon.

## PRL and vasoinhibin are endogenous players in diabetic retinopathy with translational potential

By the providing the retina and the vitreous with PRL and antiangiogenic vasoinhibin, the PRL/vasoinhibin axis contributes to the physiological restricted and avascular states of the retina and vitreous body, respectively. The natural antiangiogenic capacity of the vitreous is impaired in DR, namely by the upregulation of factors stimulating blood vessel growth, but likewise by the downregulation of inhibitors. The downregulation includes a reduced MMP-mediated conversion of PRL to vasoinhibin in DR and facilitates an increase in retinal blood vessel permeability and neovascularization growing into the vitreous, with concurrent manifestation of edema, bleeding, tractional retinal detachment, and clinically loss of vision and blindness. Preclinical experimental and clinical proof-of-concept studies revealed the translational potential of raising systemic PRL levels to elevate ocular PRL levels and enhance the generation of vasoinhibin in the vitreous. The PRL/vasoinhibin axis and its regulation in diabetes is among the factors beyond glycemic exposure which may determine the risk of DME, and DR. Therapeutic interventions are currently evaluated in a clinical trial and will show whether patients with diabetes benefit from raising circulating PRL levels. The new clinical perspective of PRL in metabolism and its contribution to the control of blood vessel growth and function *via* the PRL/vasoinhibin axis is attesting to the clinical significance of PRL beyond reproduction-associated functions.

## Author contributions

JT wrote the manuscript, TB and CC edited the manuscript. All authors approved the final version. All authors contributed to the article and approved the submitted version.

## Funding

This study was supported by the National Council of Science and Technology (CONACYT, grants 289568 and A1-S-9620B) and UNAM (grant 405PC) to CC.

## Conflict of interest

The authors declare that the research was conducted in the absence of any commercial or financial relationships that could be construed as a potential conflict of interest.

## Publisher’s note

All claims expressed in this article are solely those of the authors and do not necessarily represent those of their affiliated organizations, or those of the publisher, the editors and the reviewers. Any product that may be evaluated in this article, or claim that may be made by its manufacturer, is not guaranteed or endorsed by the publisher.
